# Olfactory mucosal mesenchymal stem cells delivered by gelatin sponge scaffolds promote functional recovery of spinal cord injury

**DOI:** 10.3389/fbioe.2025.1628758

**Published:** 2025-07-09

**Authors:** Wenshui Li, Xinchen Jiang, Shuo Lu, Wen Lu, Shanshan Ma, Yi Zhuo, Qingtao Gao, Yi Xiao, Binqian Wu, Junyang Xie, Yuhang Yu, Xiangxin Li, Que Deng, Ming Lu

**Affiliations:** ^1^ The National & Local Joint Engineering Laboratory of Animal Peptide Drug Development, College of Life Sciences, Hunan Normal University, Changsha, China; ^2^ Hunan provincical Key Laboratory of Neurorestoratology, 921 Hospital of Joint Logistics Support Force People’s Liberation Army of China (The Second Affiliated Hospital of Hunan Normal University), Changsha, China; ^3^ Department of Medical Psychology, Hunan Provincial Corps Hospital of the Chinese People’s Armed Police Forces, Changsha, China; ^4^ Department of Neurology, Clinical Medical Research Center for Stroke Prevention and Treatment of Hunan Province, Second Xiangya Hospital, Central South University, Changsha, China

**Keywords:** spinal cord injury, mesenchymal stem cell, gelatin sponge, neuroinflammation, pyroptosis

## Abstract

Spinal cord injury (SCI) is a pathological condition that damages the central nervous system. Due to the persistence of neuroinflammation after injury, the prognosis is often poor. Recent studies have found that local transplantation of mesenchymal stem cells (MSCs) can improve SCI. However, MSCs retain and engraft at the injured site limit, which may be the reason their effectiveness is greatly reduced. A gelatin sponge (GS), commonly used in clinical practice, was selected as a scaffold to deliver olfactory mucosal mesenchymal stem cells (OM-MSCs). This was done to to enhance local reparative of MSCs at the injury site. We also paid special attention to the biocompatibility of GS co-cultured with OM-MSCs *in vitro*, and then applied acellular GS and GS loaded with OM-MSCs to the rat SCI model, respectively. After the scaffold was transplanted into rat complete spinal cord injury, behavioral scores and hindlimb movement scores were improved evidently. Local inflammation in the spinal cords of transplanted rats was reduced, and the changes were related to cell pyroptosis. In addition, we found that gelatin sponges and OM-MSC transplantation did not damage other organs in rats. In conclusion, the GS scaffold loaded with OM-MSCs can reduce the local inflammatory microenvironment and facilitate neurological recovery, providing a potential and practical strategy for therapeutic approach of spinal cord injury.

## 1 Introduction

Spinal cord injury (SCI) is a severe neurological condition that causes significant motor, sensory, and autonomic dysfunction, often resulting in lifelong impairments (Dietz and Curt, 2006; Guest et al., 2022; Molinares et al., 2022; Hu et al., 2023; Tsai et al., 2024). Physically, SCI results in disabilities that affect mobility, organ function, and can lead to complications such as chronic pain, urinary tract infections, and muscle atrophy (Sun et al., 2016a; Chambel et al., 2020; Chen et al., 2022). Psychologically, SCI patients often face challenges such as depression, anxiety, and stress (Fehlings et al., 2014; Qasheesh et al., 2021).

Spinal cord tissue may be affected by hemorrhage, ischemia, glial scarring, demyelination, and remyelination following spinal cord injury (Fan et al., 2018a). Particularly after the acute phase of spinal cord injury, glial scars are formed by extremely reactive astrogliosis, astrocyte dislocation, and chondroitin sulfate proteoglycan deposition (Bradbury and Burnside, 2019). Glial scars protect surviving neurons, but they also impede nerve regeneration (Clifford et al., 2023). Several processes take place at the site of injury, including activation of astrocytes, differentiation of endogenous neural stem cells, differentiation of microglia, as well as macrophage infiltration (Stenudd et al., 2015; Yadav et al., 2023; Dougherty et al., 2000). Although the degree of neuroinflammation depends on the extent of the primary stimulus or insult, a persistent inflammatory overreaction may be the primary cause of long-term recovery difficulties (DiSabato et al., 2016). It has been shown that early intervention in the repair and regeneration of spinal cord injuries can greatly affect long-term functional recovery (Ahuja et al., 2017). Pyroptosis releases IL-1β and IL-18 and contributes to neuroinflammation after spinal cord injury (Xu et al., 2020; Chen et al., 2023), and studies have shown that inhibiting pyroptosis can improve spinal cord injury severity (Li et al., 2020; Jiang et al., 2022). Therefore, current and future treatments for SCI focus on treating neuroinflammation.

SCI treatment involves both surgical and pharmacological approaches (Silva et al., 2014; Karsy and Hawryluk, 2019). Current treatment strategies cannot completely repair SCI (Wu et al., 2022). However, partial functional neural repair has been reported in many clinical treatments or clinical trials for spinal cord injury or disorders (Huang et al., 2023a; Guo et al., 2023; Huang et al., 2022). As bioengineering technology develops, stem cells have been used to treat spinal cord injuries due to their ability to differentiate into neurons or neuronal precursor cells (Hosseini et al., 2024). By connecting the nerve fibers above and below the damaged part of the spinal cord, they form new neural circuits (Li et al., 2021). To treat spinal cord injury, cell transplantation is currently being used in many clinical trials (A. Mackay-Sim et al., 2008; Cheng et al., 2014; Sun et al., 2016b; Curtis et al., 2018; Yamazaki et al., 2020; Bydon et al., 2024), some of which use mesenchymal stem cells (MSCs), some of which use olfactory ensheathing cells (OECs), and others that use neural stem cells (NSCs). As with most MSCs, olfactory mucosa mesenchymal stem cells (OM-MSCs) exhibit strong proliferation abilities, multidirectional differentiation properties, and low immunogenicity (Ge et al., 2016). Due to their origins in the ectoderm, OM-MSCs show a strong tendency to differentiate into neurons (Yi et al., 2017; Alizadeh et al., 2019b). In comparison with Wharton’s jelly-derived mesenchymal stem cells (WJ-MSCs), OM-MSCs have a greater proliferation capacity and differentiation potential for dopamine neurons (Alizadeh et al., 2019a). OM-MSCs have a powerful repair effect in treating neurological diseases (Liu et al., 2021a; Zhuo et al., 2021; Yan et al., 2023b; Huang et al., 2024; Ge et al., 2021). Specifically, our study found that OM-MSCs can reduce neuroinflammation and alleviate microglial pyroptosis (Huang et al., 2020; Liu et al., 2021b; Zhuo et al., 2024). Based on these findings, OM-MSCs could serve as seed cells for spinal cord injury treatment.

Although local transplantation or intrathecal injection of stem cells has shown some success in treating spinal cord injury models, its limitations have limited its use. It is primarily due to the large number of transplanted cells required and the high demands on differentiation, homing, proliferation, and other characteristics. Although stem cells have a certain ability to homing, it is difficult to enrich cell suspensions in damaged areas. It may be possible to resolve this issue by combining biomaterials with cells or cell derivatives to develop biomaterial delivery systems for spinal cord injury transplantation (Han et al., 2015; Yang et al., 2020; Xu et al., 2021; Fan et al., 2022; Li et al., 2024; Pang et al., 2025b). A biomaterial’s ability to carry cells and provide structural support makes them a promising candidate for regenerating and restoring function after spinal cord injury (Fan et al., 2018b; Papa et al., 2018; Yao et al., 2018; Lv et al., 2021). Currently, the majority of spinal cord injury biomaterials are temporarily synthesized. However, even though they have been proven biocompatible and safe in animals, they have not been clinically tested. This study used medical gelatin sponge that is commonly used in clinical surgery (Kim et al., 2018b; Tsai et al., 2023). Comparing this material to other biomaterials, it has the advantages of safety, biocompatibility, loose porosity, economics, and easy accessibility (Choi et al., 1999; Ye et al., 2021).

To the best of our knowledge, this is the first study to combine medical gelatin sponge with OM-MSCs to treat spinal cord injuries. By using clinically proven materials and OM-MSCs, our research is closer to clinical application and will serve as a new reference for future regenerative treatments of spinal cord injury. By combining clinically available materials with OM-MSCs, we are bringing our research closer to clinical application, which will provide a new reference for future treatment of spinal cord injuries using regenerative medicine.

## 2 Materials and methods

### 2.1 Isolation and cultivation of OM-MSCs

OM-MSCs were obtained from healthy volunteers for scientific research purposes at the Second Affiliated Hospital of Hunan Normal University with ethical approval. All experimental protocols were approved by the Biomedical Research Ethics Committee of Hunan Normal University (approval number 2021–347) and were performed in accordance with the ethical guidelines of the World Medical Association (Declaration of Helsinki). Fresh nasal mucosal tissue was obtained after obtaining signed informed consent from all donors. Three days before surgery, volunteers trimmed their nose hair and used chloramphenicol nasal drops three times a day. As part of the preparation for surgery, the patient’s nasal cavity was thoroughly cleaned and disinfected, and tetracaine was used as a local anesthetic. As soon as the anesthesia is complete, remove about 5 mm^3^ of superior nasal turbinate tissue and immediately place it in normal saline. Nasal mucosal tissue was cut into 1 mm^3^ cubes and seeded into 25 cm^2^ culture flasks with standard culture medium consisting of DMEM/F12 (Gibco, Carlsbad, CA, United States) and 10% heat-inactivated fetal bovine serum (FBS, Gibco, Carlsbad, CA, United States). The culture medium was changed every 3 days and maintained at 37°C, 5% CO_2_, and 95% humidity. There were a large number of OM-MSCs crawling out of the tissue blocks after 4 weeks of culture. The OM-MSCs were obtained after 5 min of digestion with 0.1% trypsin, the cell is recorded as generation 1. Cells were grown in an incubator at 37°C, 5% CO_2_, and 95% humidity, with the medium replaced every 3 days. As soon as the cell confluence reached 80%–90%, the OM-MSCs were digested with 0.25% trypsin-EDTA (Gibco, Carlsbad, CA, United States) for 30 s, and digestion was terminated with complete medium containing fetal bovine serum. A ratio of 1:4 was used to subculture the cells, and high-purity OM-MSCs were obtained following the fourth generation.

### 2.2 Flow cytometric identification of OM-MSCs

Preparation of the fourth generation OM-MSCs into cell suspension, add 100ul of cell suspension to each tube, and then add 2ul of CD34, CD45, CD73, CD90, CD105 antibodies (Invitrogen, Waltham, MA, United States) to stain the cells, add anti-IgG-PE (Invitrogen, Waltham, MA, United States) to the negative control tube, and incubate in a 4°C refrigerator away from light for 30 min. Wash the cells twice with PBS, and then put the samples into the flow cytometer for detection. It was found that the purity of the OM-MSCs was greater than 98%.

### 2.3 Adipogenesis differentiation of OM-MSCs

Inoculate 5 × 10^4^ cells into the wells of a 6-well plate with added culture medium. After the cells have attached, replace media with pre-warmed complete adipogenesis differentiation medium (Gibco, Carlsbad, CA, United States) and continue incubation. Each well of the six-well plate was filled with 2 mL of differentiation medium, which was replaced every 3 days according to the manufacturer’s instructions. Three weeks after cultivation, remove the culture medium and clean with PBS three times. 2 mL of 4% paraformaldehyde solution should be added to each well of the six-well plate, and the solution should be fixed for 10 min, followed by three washes with PBS. For staining, add 2 mL of Oil Red O dye working solution to each well and incubate for 30 min at room temperature. After three washes with PBS to remove excess dye, the specimens were observed under a light microscope.

### 2.4 Osteocyte differentiation of OM-MSCs

Inoculate 5 × 10^4^ cells into the wells of a 6-well plate with added culture medium. After the cells have attached, replace media with pre-warmed complete osteogenesis differentiation medium (Gibco, Carlsbad, CA, United States) and continue incubation. Each well of the six-well plate was filled with 2 mL of differentiation medium, which was replaced every 3 days according to the manufacturer’s instructions. 21 day after cultivation, remove the culture medium and clean with PBS three times. 2 mL of 4% paraformaldehyde solution should be added to each well of the six-well plate, and the solution should be fixed for 10 min, followed by three washes with PBS. For staining, add 2 mL of Alizarin Red Staining Solution to each well and incubate for 10 min at room temperature. After three washes with PBS to remove excess dye, the specimens were observed under a light microscope.

### 2.5 Preparation of gelatin sponge loaded with OM-MSCs

15 × 15 × 5 mm^3^ gelatin sponge was washed 3 times with PBS in advance and immersed in 0.75 mg/L PLL solution (Beyotime, Shanghai, China) at room temperature overnight. After washing 3 times with PBS, the gelatin sponge was placed in the center of a six-well plate, and 1 mL of complete culture medium containing 1 × 10^6^ OM-MSCs was evenly inoculated on the surface of the gelatin sponge and incubated in a humidified 5% CO_2_ at 37°C for 2 h. Subsequently, 2 mL of complete culture medium was added to the six-well plate, and the morphological changes of the gelatin sponge were photographed with a mobile phone during the entire experiment.

### 2.6 CCK-8 cell activity assay

When OM-MSCs were cultured in gelatin sponge for 1, 3, 5, 10, 15, 20, 25 and 30 days, the cell supernatant was aspirated, CCK-8 reagent (Beyotime, Shanghai, China) and complete culture medium were mixed in a ratio of 1: 9, and 2 mL was added to a 6-well plate. After gently shaking, the plate was incubated in a humid 5% CO_2_ at 37°C for 0.5 h, and then the supernatant was aspirated and transferred to 100 μL in a 96-well plate, with 6 replicate wells in each group; the 96-well plate was placed in an reader to detect the absorbance value at an excitation wavelength of 450 nm, and the cell viability was calculated.

### 2.7 ELISA

The levels of lactate dehydrogenase (LDH), interleukin-1 beta (IL-1β) and tumor necrosis factor-alpha (TNF-α) in spinal cord tissues from Sprague-Dawley (SD) rats were quantified using an ELISA kit (4A Biotech, Suzhou, China). After incubation with a working solution, samples were exposed to a substrate solution at 37°C for 0.5 h. After adding the termination solution, the absorbance was measured using a microplate reader (Thermo Fisher, United States).

### 2.8 Live and dead cell staining

Discard the supernatant of the corresponding days of gelatin sponge and wash it twice with PBS. Use Calcein-AM/PI live/dead cell double staining kit (Solarbio, Beijing, China) to stain the cells. Follow the instructions and incubate in a cell culture incubator. After incubation, aspirate the staining solution, add PBS, and observe and photograph the live and dead conditions of OM-MSCs in the gelatin sponge under a fluorescence microscope.

### 2.9 Animal and SCI models

All animal experiments were performed in accordance with the guidelines approved by the Biomedical Research Ethics Committee of Hunan Normal University (approval number 2021–347). Female Sprague Dawley rats (200–220 g), 8 weeks old, were purchased from Hunan SJA Laboratory Animal Co., Ltd. (Changsha, China). All rats were housed in a temperature-controlled room (22°C ± 1°C) with 12 h light/12 h dark cycle. Forty female SD rats were randomly divided into sham operation group, SCI group, GS group, and GS + OM-MSCs group, with 10 rats in each group. We established a rat model of completely transected SCI, with a 28-day observation period after modeling. An intraperitoneal injection of sodium pentobarbital (50 mg/kg) anesthetized the rats, and the target T10 vertebra was located through the ribs, an incision was made with a scalpel, and the surrounding tissue of the vertebra was bluntly separated. The muscles were cut and separated layer by layer to expose the T10 lamina and spinous process. The spinous process and lamina were only bitten off with bone rongeurs to expose the spinal cord, and the remaining bone structures and intact dura mater were preserved. To establish a completely transected spinal cord injury, the SCI group, GS group, and GS + OM-MSCs group used ophthalmic scissors to remove 3 mm columnar spinal cord tissue. The Sham group was left untreated except for laminectomy. Both the GS and GS + OM-MSCs groups had the spinal cord defects filled with gelatin sponges, followed by layering sutured muscles, fascia, and skin, and disinfected external skin. Postoperatively, the animals were resuscitated in an incubator and provided with adequate food and water. Penicillin (2 × 10^6^ U/kg/day) was administered for 3 days to prevent infection, and the bladder was massaged twice daily until the bladder function was restored. Record the daily survival status of each group and plot the overall survival curve.

### 2.10 Behavioural assessment

After surgery, functional recovery following SCI was evaluated utilizing the Basso, Beattie, and Bresnahan (BBB) assessment and footprinting. Two blinded investigators evaluated sensory and motor functions on postoperative days 0, 7, 14, 21, and 28 in an unconfined setting. The assessment scale, spanning from 0 (representing paralysis of the whole body) to 21 (representing full functional recovery), was recorded independently by each investigator, and the composite score was derived by computing the mean of the evaluations. From each group, five surviving rats were randomly selected and marked with blue and red ink on their forelimbs and hindlimbs, then allowed to walk through a path lined with white paper. Two impartial observers independently examined the footprints without knowing the parameters of the experiment.

### 2.11 Histological analysis

Immunohistochemistry was used to evaluate the day 28 spinal cord. On the day of sacrifice, rats were deeply anesthetized and perfused intracardially with PBS followed by 4% paraformaldehyde (PFA). The spinal cord was then removed, fixed in 4% PFA for 24 h, embedded in paraffin, and cut into serial longitudinal sections with a thickness of 10 μm using a cryostat (LEICA RM2125 RTS, Leica Biosystems, Nussloch, Germany).

Hematoxylin-eosin (H&E) staining for evaluation of spinal cord tissue structure. The sections of brain sections were deparaffinized with graded ethanol together with xylene, and 4 μm sections were prepared for HE staining.

Nissl staining for assessment of neuronal damage. Cresyl violet (Beyotime, Shanghai, China) was used to stain brain sections for 30 min at room temperature. Next, brain slices were washed with distilled water, treated with 95% ethanol for 30 s, covered with 50% glycerol, and dried.

Immunofluorescence staining was performed on day 28 specimens to evaluate pyroptosis. Briefly, Spinal cord sections were incubated overnight at 4°C with NLRP3 (Abcam, Fremont, CA, United States) and Caspase-1 (Abcam, Fremont, CA, United States) and then with Alexa Fluor 594 goat anti-mouse (Beyotime, Shanghai, China). Mounting medium containing DAPI (Beyotime, Shanghai, China) was used to visualize nuclei.

### 2.12 Western blotting

The tissues were processed for Western blot analysis. We lysed the samples using RIPA lysis buffer (Beyotime, Shanghai, China). We followed the instructions according to the instructions provided by the manufacturer. We determined the protein concentration using a BCA protein assay kit (Beyotime, Shanghai, China). Each group of proteins from the adjusted samples were loaded onto the gel and the proteins were transferred to a polyvinylidene fluoride (PVDF) membrane. The membranes were then blocked with 5% skim milk for 1 h and incubated with primary antibodies overnight at 4°C. The antibodies used in this study included NLRP3 (dilution 1:1,000, 27458-1-AP, Proteintech, China), Caspase-1 (dilution 1:1,000, ab207802, Abcam, United States), GSDMD (dilution 1:2000, ab209845, Abcam, United States), β-actin (dilution 1:5,000, 10663-1-AP, Proteintech, China), IL-1β (dilution 1:1,000, bs-0812R, Bioss, United States) and IL-18 (dilution 1:2000, 27458-1-AP, Proteintech, China). After three washes with TBST, the membranes were incubated for 1 h at room temperature with HRP-conjugated secondary antibodies. Finally, imaging was achieved using ECL chemiluminescence substrate (BL520B; Biosharp, China), and the intensity of the bands was quantified using ImageJ software.

### 2.13 Electron microscope test

The collected SD rat spinal cord or Gelatin sponges were fixed overnight at 4 °C with 2.5% glutaraldehyde, followed by washing in cacodylate buffer and fixation with 1% osmium tetroxide. After another round of washing in cacodylate buffer, the samples were embedded in a double-staining solution containing 5% uranyl acetate and lead citrate before being sectioned using an ultramicrotome for examination under an electron microscope (HT7700, Hitachi, Tokyo, Japan).

### 2.14 Monitoring the survival of donor cells

OM-MSCs were labeled with GFP (Genechem, Shanghai, China) in advance. SCI rats were imaged using the IVIS Lumina II imaging system 28 days after surgery. Analysis was performed based on regions of interest (ROI).

### 2.15 Statistical analysis

We present the data as a mean ± standard deviation (SD). Student’s t-tests,or oneway analysis of variance (ANOVA) with the Bonferroni corrections for post hoc t-test, or two-sided ANOVAs with Bonferroni corrections for post hoc t-tests were conducted to estimate differences between groups. GraphPad Prism 10 Software was used for the statistical analysis (La Jolla, CA, United States). Each experiment was replicated at least three times. P-values less than 0.05 were considered significant.

## 3 Results

### 3.1 Changes in the morphology and cell viability of gelatin after OM-MSCs were bound to gelatin sponges

**FIGURE 1:**
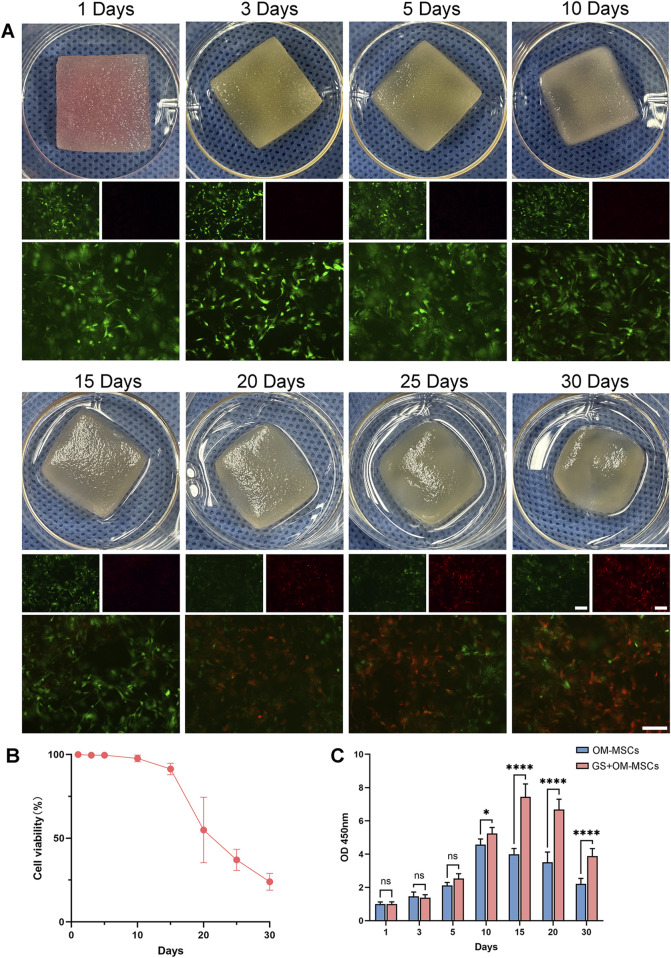
Gelatin sponge morphology and cell viability after OM-MSC loading. **(A)** Gelatin sponge morphology and live-death staining within 30 days. Green indicates living cells and red indicates dead cells. Photo scale bars = 10 mm and fluorescent images scale bars = 50 μm. **(B)** Analysis of gelatin sponge cell viability with live-dead staining. Data are shown as mean ± SD, N = 3. **(C)** OM-MSCs cultured conventionally and OM-MSCs loaded on gelatin sponge were tested for cell viability with CCK-8. Data are shown as mean ± SD, N = 3 (*p < 0.05, ****p < 0.0001, ns non-significant).

**FIGURE 2:**
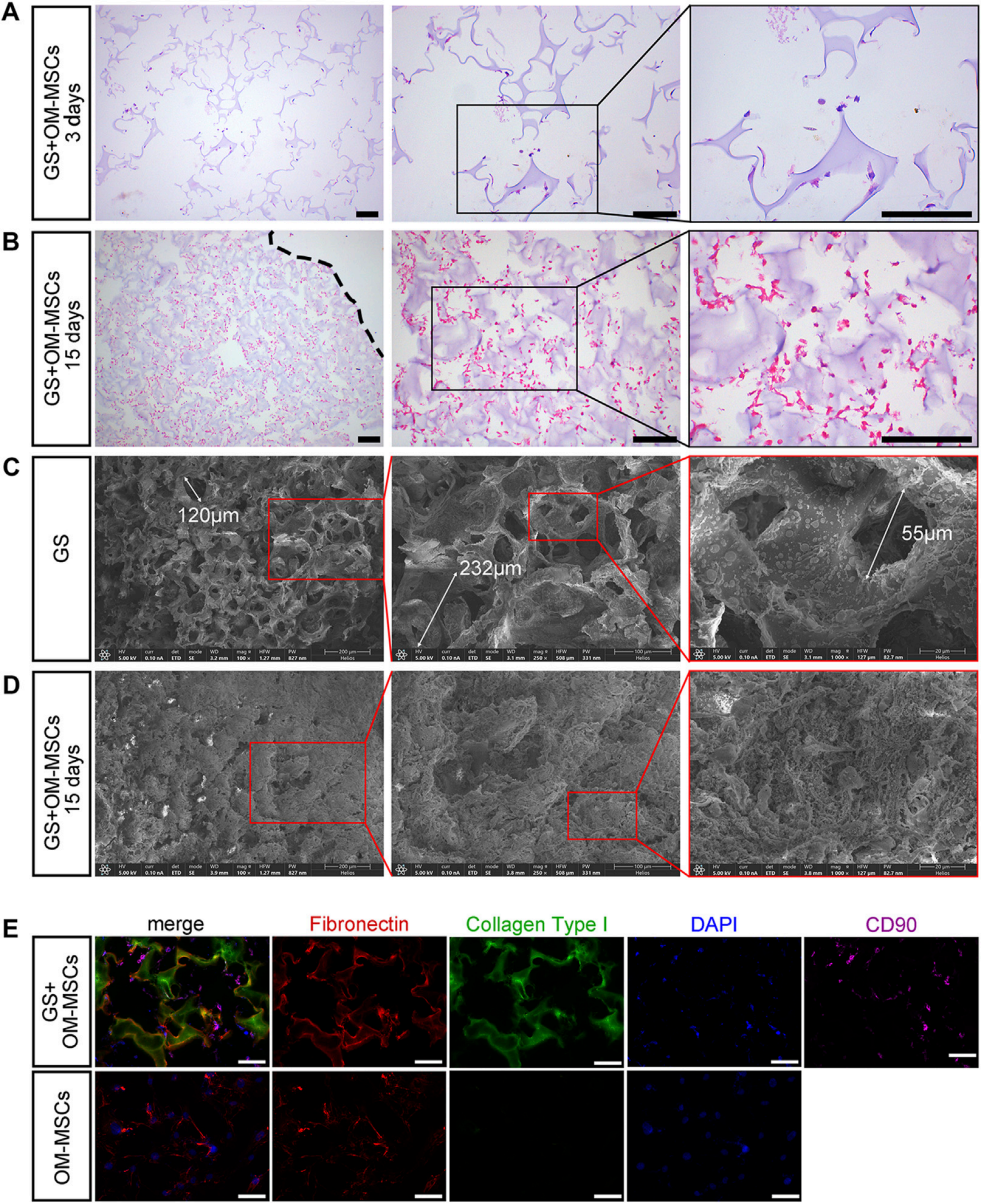
The histological changes caused by loaded gelatin sponges with OM-MSCs. HE staining of gelatin sponge loaded with OM-MSCs on days 3 **(A)** and days 15 **(B)**. A dotted line indicates the surface of the gelatin sponge. Scale bars = 100 μm. Scanning electron microscopy of gelatin sponge without or with OM-MSCs **(C)** for 15 days **(D)**. **(E)** OM-MSCs and GS + OM-MSCs stained with extracellular matrix markers and OM-MSC markers. Scale bars = 50 μm.

The OM-MSCs were isolated and purified according to previously described protocols (Zhuo, 2024), and their cell surface markers and differentiation capacity were characterized (Supplementary Figure S1). 1 × 10^6^ OM-MSCs were seeded in a 15 × 15 × 5 mm^3^ gelatin sponge, and their morphology was observed. As the culture time increased, the gelatin sponge size decreased. After 10 days of culture, the edges gradually became rounded and the transparency became higher and higher. Gelatin sponges showed jelly-like properties after 20 days of culture. Early in the culture process, OM-MSCs were sparsely distributed in the gelatin sponge (Figure 2A). After 15 days of culture, the OM-MSCs were evenly distributed throughout the material, and the number of OM-MSCs increased (Figure 2B). After 30 days of culture, the distribution of OM-MSCs in the gelatin sponge significantly changed, and the number of cells distributed on the surface was significantly greater than that in the center (Supplementary Figure S2A). It is possible that this is the reason why the center of the gelatin sponge collapsed after 30 days of culture (Figure 1A).

### 3.2 Gelatin sponge and OM-MSC culture strategy

We performed live-dead staining on OM-MSCs in gelatin sponges within 30 days of culture to determine the optimal culture time for OM-MSCs (Figure 1A). On the gelatin sponge, OM-MSC viability declined after 10 days, and a large number of cells died after 15 days (Figure 1B). Under the same culture conditions, conventionally cultured OM-MSCs and OM-MSCs loaded on gelatin sponge were compared for cell viability. A CCK-8 study showed that conventionally cultured OM-MSCs slowed down after 5 days, reaching peak viability on day 10. In contrast, OM-MSCs loaded on gelatin sponge reached their peak viability on day 15, showing a better cell proliferation effect than conventional culture (Figure 1C). We chose 15 days as the duration for culturing OM-MSCs in the gelatin sponge since cells cultured for 10–15 days have a strong proliferation ability and have a relatively small number of dead cells. Compared with conventional 2D culture, gelatin sponge can provide OM-MSCs with an environment that enhances cell viability, which is one of its advantages.

### 3.3 Histological changes of OM-MSCs after binding to gelatin sponges

In comparison with the 3 days culture group, OM-MSCs grew in the gaps of the gelatin sponge after 15 days of culture (Figures 2A,B). Based on scanning electron microscopy (SEM) observations (Figure 2C), the gelatin sponges without OM-MSC growth had a loose and porous structure with pores between 50 and 300 μm, which enabled mesenchymal stem cells to gro. The gelatin sponge was filled with OM-MSCs after 15 days of growth (Figure 2D), indicating good biocompatibility. OM-MSCs grown for 30 days on gelatin sponge showed no significant increase in cell number and many fragmented nuclei, indicating cell death after HE staining and SEM (Supplementary Figure S2A). In order to further investigate the biocompatibility of gelatin sponge with OM-MSCs, we detected markers of extracellular matrix. OM-MSCs, laminin, fibronectin and cell nuclei were labelled after 15 days of gelatin sponge culture and laminin and fibronectin expression increased significantly (Figure 2E). A biocompatible combination of OM-MSC and gelatin sponge is demonstrated here.

### 3.4 Gelatin sponges containing OM-MSCs improve motor function in rats with spinal cord injuries

**FIGURE 3:**
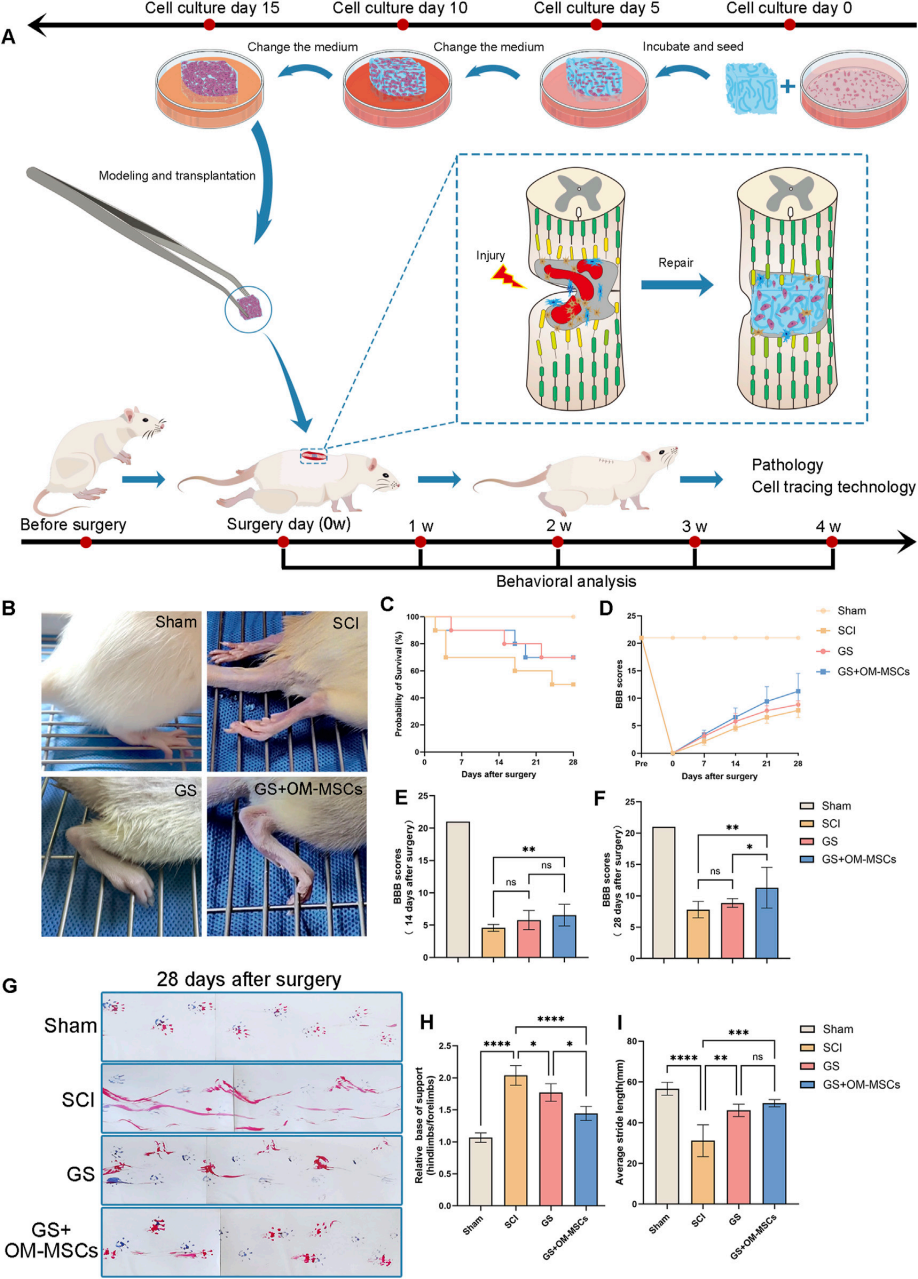
Gelatin sponges containing OM-MSCs improve motor function in rats with spinal cord injuries. **(A)** Schematic diagram of the experimental procedure. **(B)** The hindlimb images of SCI rats at 28 days after different treatments. **(C)** Survival curves of rats in each group. **(D)** Following surgery, the BBB scores of SD rats changed in each group over the following 28 days. SD rats at 14 days **(E)** and 28 days **(F)** after surgery with BBB scores. 28 days after surgery, gait analysis images **(G)**, relative base of support **(H)**, and average stride length **(I)** were taken of SD rats. Data are shown as mean ± SD, N = 4 (*p < 0.05, **p < 0.01, ***p < 0.001, ****p < 0.0001, ns non-significant).

It is common for incomplete spinal cord injuries to result in better recovery of motor function, but this is not necessarily a result of the spinal cord tissue healing on the injured side (Friedli et al., 2015). Therefore, we performed a complete spinal cord injury model on the T10 segment of 40 SD female rats. We performed local transplantation of cell-free gelatin sponges or OM-MSC-loaded gelatin sponges immediately after modeling (Figure 3A). After modeling, motor function evaluations and pathological analyses were performed on the Sham group (only the spinal cord was exposed), the SCI group (the spinal cord was completely cut), the GS group (the spinal cord was completely cut and gelatin sponge was transplanted), and the GS + OM-MSCs group (the spinal cord was completely cut and gelatin sponge loaded with OM-MSCs was transplanted).

As inflammation often occurs in the early stages of spinal cord injury and affects repair later, we set a 4-week observation period. 28 days after surgery, the hind limb performance of the rats in each group was as follows: The rats treated with GS were able to walk using plantar, whereas SCI group rats were not (Figure 3B). In the early period after surgery, particularly in the SCI group, rats had a relatively high mortality rate, as shown by the survival curve (Figure 3C). On the day of SCI modeling and 1–4 weeks after modeling, SD rats were scored for Basso–Beattie–Bresnahan (BBB) scores to determine the long-term effect of OM-MSCs on motor recovery (Figure 3D). The pre-modeling score of each group was 21 points, and on the day of SCI modeling, the total score of the rats who underwent complete spinal cord injury modeling was 0 points, and the average BBB score gradually increased in the following 1–4 weeks 14 days after surgery, the BBB scores of rats in the GS + OM-MSCs group were higher than those in the SCI group. However, there was no significant difference between the GS group and the SCI group (Figure 3E). 28 days after surgery, the BBB score showed that the GS + OM-MSCs group was significantly higher than the GS group. However, there was no significant difference between the GS group and the SCI group (Figure 3F). The results showed that transplantation of gelatin sponges was helpful for functional recovery in rats with spinal cord injury. The repair rate was significantly reduced with time, but gelatin sponges loaded with OM-MSCs continued to have functional repair effects.

28 days after surgery, we performed gait analysis in each group of rats. We smeared the rat forefoot with blue ink and the rat forefoot with red ink, then passed on a runway with a length of 600 mm and a width of 100 mm (Figure 3G). As the gait diagram shows, the hindlimbs of the SCI group dragged mostly, whereas the GS and GS + OM-MSC groups dragged less, and the footprints of the GS + OM-MSC group were clearer. According to the results of gait analysis, we counted the relative area of the hind limbs and forefoot of the rats in each group, and found that the relative areas of the hind limbs in the GS + OM-MSCs group and the GS group were lower than those in the SCI group, and the contact area of the hind limbs in the GS + OM-MSCs group decreased the most significantly (Figure 3H). In gait analysis, the average stride length was significantly greater in the GS group and the GS + OM-MSCs group than in the SCI group, but the difference in stride length between the GS group and the GS + OM-MSCs group was not significant, probably due to similar degrees of recovery in the large joints of the hind limbs (Figure 3I). SCI rats can walk better after receiving gelatin sponge transplants, and OM-MSCs can enhance this effect.

### 3.5 Gelatin sponge scaffolds delivering OM-MSCs improves spinal cord tissue architecture and reduces inflammation in the spinal cord

**FIGURE 4:**
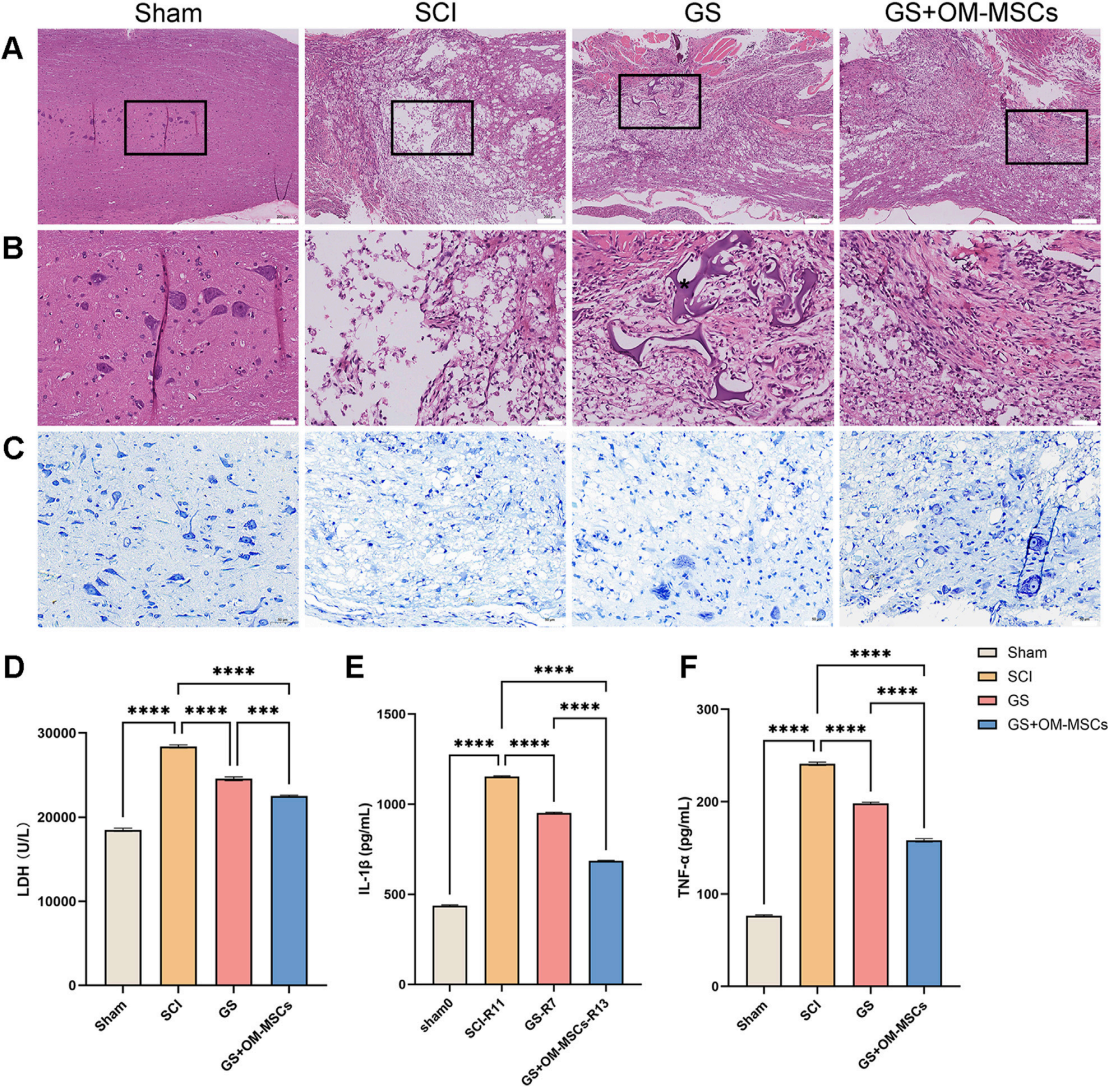
Spinal cord tissue effects of gelatin sponge combined with OM-MSCs. **(A)** Staining of the spinal cord injury area with HE. Scale bars = 200 μm. **(B)** HE staining of the spinal cord injury’s central area. *: gelatin sponges. Scale bars = 50 μm. **(C)** Nissl staining of the spinal cord injury’s central area. Scale bars = 50 μm. Tissue LDH **(D)**, IL-1 **(E)**, and TNF-α **(F)** levels were measured by ELISA. Data are shown as mean ± SD, N = 4 (***p < 0.001, ****p < 0.0001).

According to histological examination of the SCI group, an infiltrating cavity appeared in the middle of the lesion, with inflammatory cells infiltrating it, and evident vacuoles on both sides of the lesion, suggesting that the inflammation is significant. Comparatively, spinal cord tissue lesions were milder in the GS group and GS + OM-MSCs group (Figure 4A). Probably, the spinal cord tissue material supports tissue regeneration after spinal cord injury, which promotes nerve cell growth. Interestingly, in the spinal cord section, we found unabsorbed gelatin sponge material encased in the spinal cord tissue (Figure 4B). GS + OM-MSCs had similar tissue structure to GS, but there was no unabsorbed gelatin sponge material in the sections. Since OM-MSCs were cultured with gelatin sponge for 15 days *in vitro*, the cells and sponge tissue have merged, and they are more easily absorbed after being transplanted into rat spinal cord tissue, which demonstrates gelatin sponge’s tissue compatibility.

Nissl staining revealed few Nissl bodies in the spinal cords of rats with spinal cord injuries, indicating severe nerve damage (Figure 4C). In the GS group, only a small number of neuronal cell bodies were observed; in the GS + OM-MSCs group, neuronal cell bodies increased in number and size, suggesting that the transplanted gelatin sponge and OM-MSCs protected neurons.

Through ELISA detection, we further analyzed the levels of related inflammatory factors in the tissues. The levels of LDH, IL-1β, and TNF-α were highest in the SCI group, while these inflammatory markers were further reduced in the GS group and the GS + OM-MSCs group (Figures 4D–F). Based on the above, we found that the transplantation of gelatin sponges could alleviate the neuroinflammation of a part of the spinal cord tissue. The effect of gelatin sponges loaded with OM-MSC was more obvious.

### 3.6 Gelatin sponge scaffolds delivering OM-MSCs protect neurons from pyroptosis and reduce neuroinflammation in SCI rats

**FIGURE 5:**
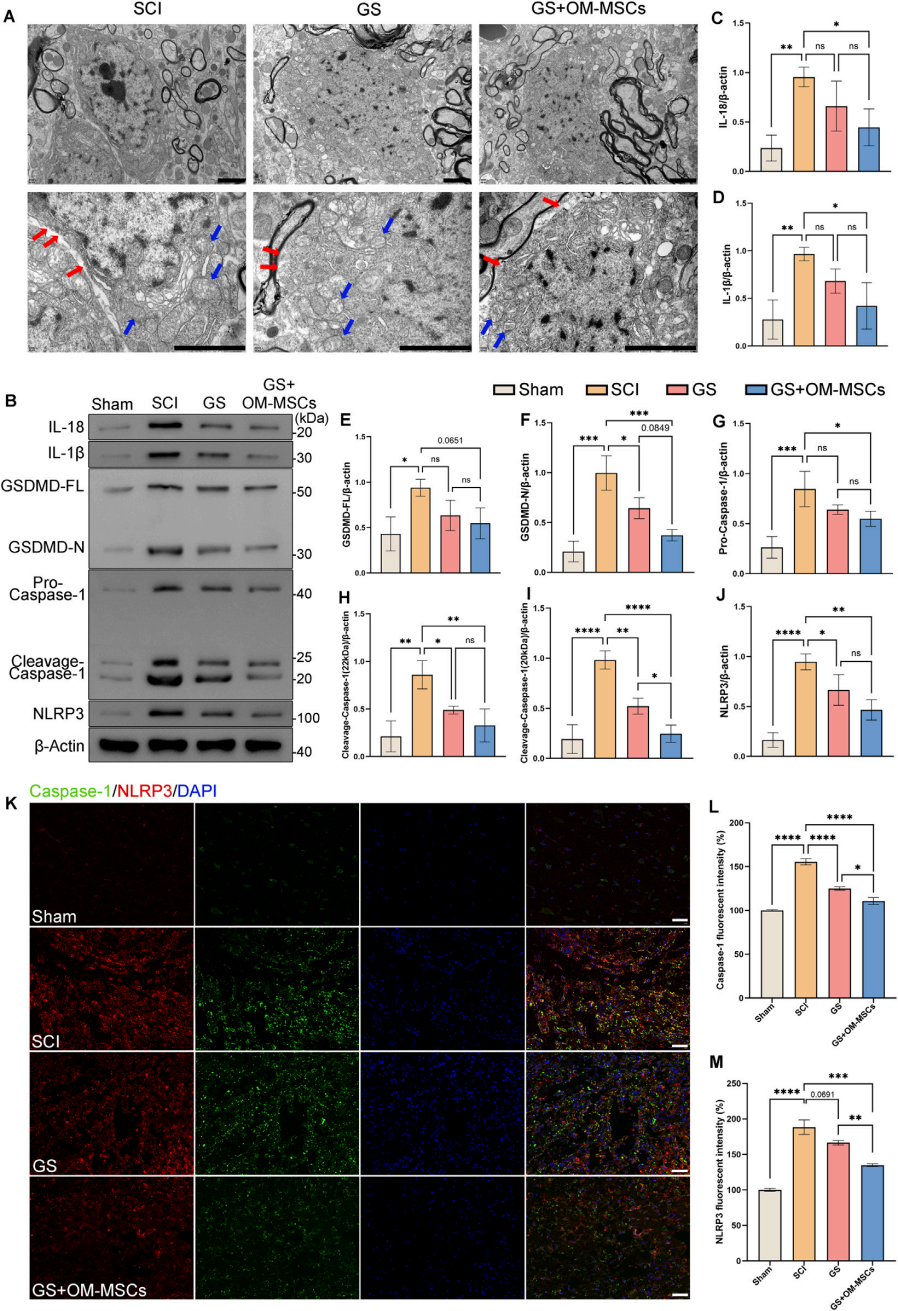
The GS and OM-MSCs inhibit pyroptosis and reduce neuroinflammation in spinal cord injured rats. **(A)** Neuronal structure is revealed by TEM. Red arrow: Broken cell membrane. Blue arrows: swollen mitochondria. Scale bars = 2 μm. **(B)** Western blot of the spinal cord tissue. Western blot analysis of the protein levels of IL-18 **(C)**, IL-1β **(D)**, GSDMD **(E,F)**, Caspase-1 **(G–I)** and NLRP3 **(J)** in spinal cord tissue, N = 3. **(K)** Injured areas of the spinal cord are stained with pyroptosis markers. Green: Caspase-1, Red: NLRP3, Blue: DAPI. Scale bars = 50 μm. **(L)** Caspase-1 fluorescence intensity statistics, N = 4. **(M)** NLRP3 fluorescence intensity statistics, N = 4. Data are shown as mean ± SD (*p < 0.05, **p < 0.01, ***p < 0.001, ****p < 0.0001, ns non-significant).

According to histology and related detection of inflammatory factors, the local lesions of spinal cord injury rats are in a state of inflammation for a long time, leading to the death of a large number of neurons, which may be an important reason affecting their functional recovery (DiSabato et al., 2016; Orr and Gensel, 2018; Pang et al., 2021). The cellular structure of neurons and microglia in spinal cord tissue from SCI rats was examined using TEM. There were enlarged neuronal cell bodies, multiple discontinuities in cell membranes, vacuoles in the cytoplasm, enlarged mitochondria, and swollen mitochondrial cristae, all suggesting that damage had been done to organelles such as mitochondria, endoplasmic reticulum, and Golgi apparatus (Figure 5A). The SCI group showed multiple holes in microglia, disordered cytoplasm, and swollen mitochondria (Supplementary Figure S3). The typical cell structural features suggest that the cells are undergoing pyroptosis. IL-1β and IL-18 can also be released due to cell pyroptosis, increasing the inflammatory response. Therefore, we speculate that the inflammatory response in spinal cord injury is related to pyroptosis.

In pyroptosis, proteins such as IL-1β, IL-18, GSDMD, Caspase-1, and NLRP3 cooperate to enhance inflammatory responses and death of cells. As a core component of the inflammasome, NLRP3 detects intracellular danger signals and initiates the pyroptosis signaling cascade. WB detection showed that the NLRP3 protein level in the SCI group was significantly increased (Figures 5B,J). However, the protein content of the pyroptosis-related NLRP3 inflammasome was significantly reduced in the GS group and the GS + OM-MSCs group.

The NLRP3 inflammasome can further activate Pro-Caspase-1 to become active Cleavage-Caspase-1 (Swanson et al., 2019). As shown in the test results (Figures 5B,G,H,I), the level of Pro-Caspase-1 is increased in the SCI group, whereas it is not significantly reduced in the GS group, only in the GS + OM-MSCs group. Further, the GS group and the GS + OM-MSCs group showed significantly reduced levels of Cleavage-Caspase-1 (22 kDa) and Cleavage-Caspase-1 (20 kDa).

Gasdermin D (GSDMD) is a pyroptosis execution protein (Shi et al., 2017). Caspase-1 or Caspase-4/5/11 cleave GSDMD to generate active N-terminal fragments in response to intracellular inflammation. The N-terminal fragment of GSDMD can insert into the cell membrane and form pore-forming, which damages the integrity of the cell membrane, causing cell contents leakage and triggering pyroptosis. The test results showed that the full-length GSDMD in the SCI group was significantly increased, but the levels in the GS group and GS + OM-MSCs group were not substantially reduced. On the contrary, the level of active GSDMD-N was significantly reduced in the GS group and the GS + OM-MSCs group (Figures 5B,E,F).

Pyroptosis induces an inflammation dependent on IL-1β and IL-18 (Chen et al., 2023). Through the membrane pores formed by GSDMD, IL-1β and IL-18 can be rapidly released into the extracellular space, further prolonging the inflammatory response. From the test results, it can be seen that the levels of IL-1β and IL-18 in the SCI group were significantly increased, and the levels in the GS group were not significantly reduced, but the levels in the GS + OM-MSCs group were considerably lower than those in the SCI group (Figures 5B–D).

The activation of GSDMD causes the degradation of membranous organelles such as mitochondria in pyroptotic neurons and irreversible damage to the plasma membrane around the ischemic region (Sborgi et al., 2016). As compared to the SCI group, GS and GS + OM-MSCs significantly reduced the rupture of the membrane, cytoplasmic disorder, and mitochondrial swelling in neurons. The GS group and GS + OM-MSCs group also showed improved cell structure and organelle morphology than the SCI group. Moreover, mitochondrial swelling and cytoplasmic disorder were reduced further in the GS + OM-MSC group, and more normal mitochondria and organelles were retained (Figure 5A).

A study of immunohistofluorescence in spinal cord tissue revealed increased expression of Caspase-1 and NLRP3 in the SCI group, while after gelatin sponge transplantation, pyroptosis-related markers were downregulated, especially in the GS + OM-MSCs group, which was more effective at inhibiting pyroptosis (Figures 5K–M). According to the results of the above tests, gelatin sponges and OM-MSC transplantation have been shown to inhibit pyroptosis in rats following spinal cord injury.

### 3.7 Biocompatibility of gelatin sponges combined with OM-MSCs in rats

**FIGURE 6:**
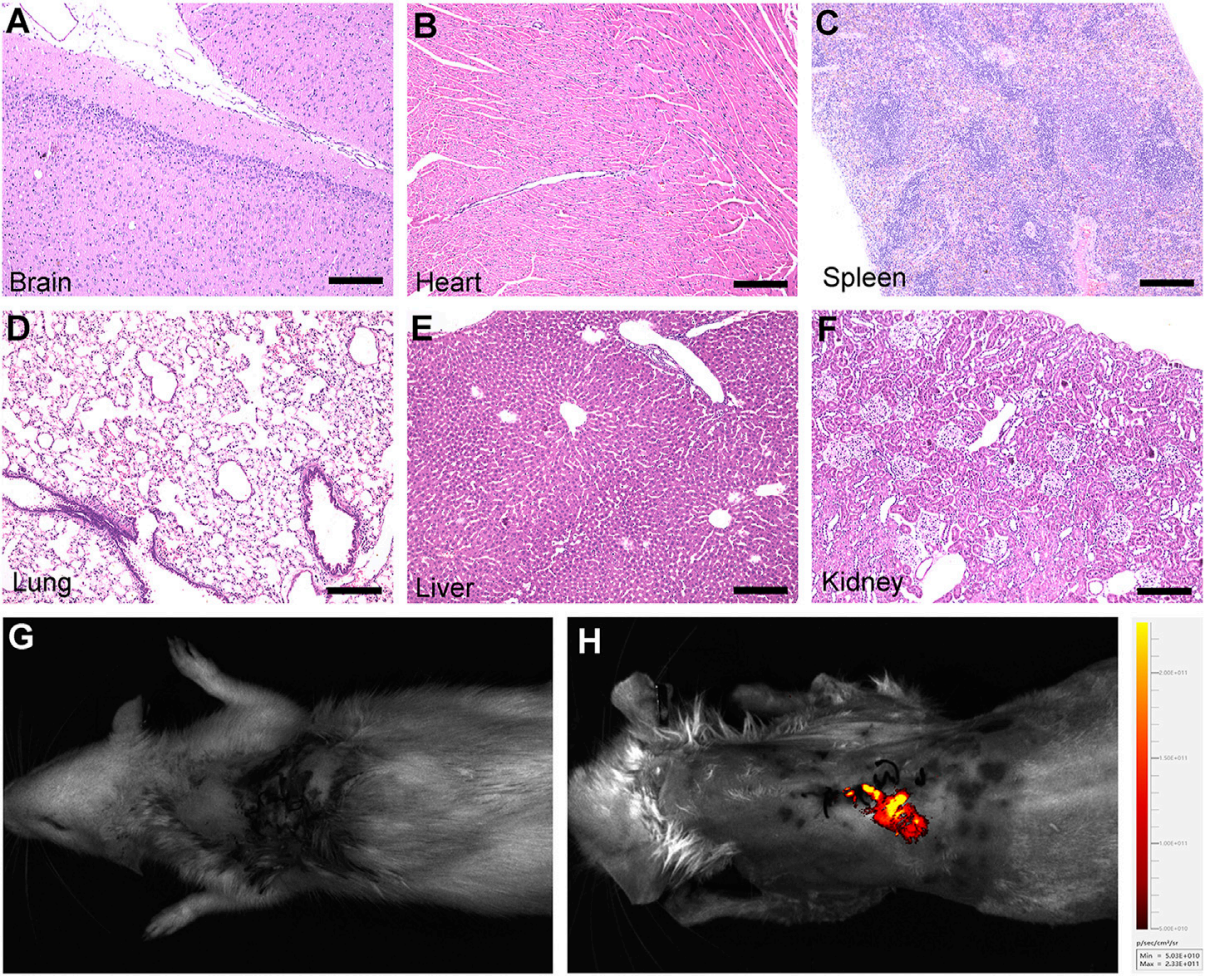
Biocompatibility of gelatin sponge combined with OM-MSCs in rats. **(A-F)** HE staining of brian, heart, spleen, lung, liver and kidney structures after transplantation in the GS + OM-MSCs group. **(G,H)** The fluorescence signal of GPF-labeled OM-MSCs is shown in Live Animal Imaging. Scale bars = 200 μm.

28 days after the OM-MSCs loaded with gelatin sponge were transplanted into SCI rats, HE staining analysis was performed on multiple organs of the rats. The results showed that the rats’ brain, heart, spleen, lung, liver, and kidney tissue structures were normal 28 days after transplantation (Figures 6A–F). In order to observe whether OM-MSCs can survive *in vivo*, we used green fluorescent protein to mark OM-MSCs in advance, and used small animal *in vivo* imaging to observe the fluorescence signal of OM-MSCs at the surgical site of the rats 28 days after transplantation (Figures 6G,H). GS + OM-MSCs group displayed obvious GFP fluorescence signals at the surgical site. In short, the above results show that OM-MSCs can survive in rats, and gelatin sponge and OM-MSCs have advantages in biocompatibility.

## 4 Discussion

The main reasons why spinal cord injury is difficult to fully recover include the non-regenerative nature of neurons, glial scar formation, inflammation response, axon growth inhibition environment, lack of neurotrophic factors, vascular damage and ischemia, cell apoptosis, and difficulty in neural circuit reconstruction. These factors can also be targeted as treatment targets.

**FIGURE 7:**
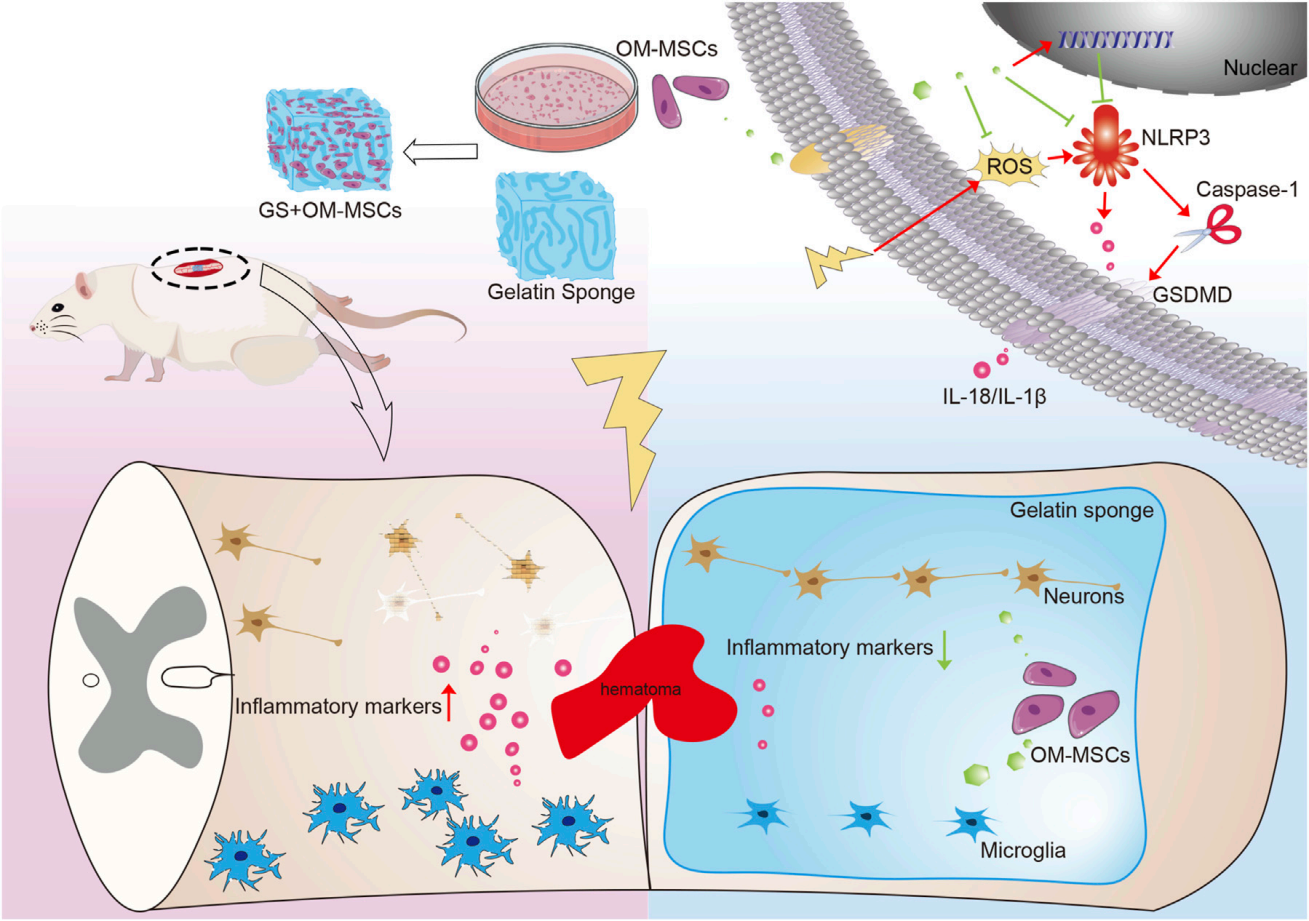
Schematic diagram of the mechanism by which gelatin sponge scaffolds deliver OM-MSCs affected functional recovery of spinal cord injury. The gelatin sponge delivery system carrying OM-MSCs reduces the local inflammatory environment by reducing pyroptosis, repairs tissue structure, and promotes neurological function recovery in spinal cord injury.

In this study, we loaded OM-MSCs onto gelatin sponge materials and directly filled them into the spinal cord defect site. This proved that OM-MSCs and gelatin sponges inhibit pyroptosis at the injury site, regulate neuroinflammation, and protect nerve function (Figure 7). There are three pieces of evidence to support this view. First, OM-MSCs and gelatin sponges can significantly reduce inflammatory factors in spinal cord tissue and downregulate pyroptosis. Second, treatment with OM-MSCs and gelatin sponges restored the structure of injured spinal cord tissue. Finally, after transplantation treatment in rats with spinal cord injury, impaired motor ability was significantly restored. This highlights the significant role of OM-MSCs and gelatin sponges in protecting nerve function.

Gelatin sponges are well known as auxiliary material, mainly used to treat hemostasis, promote wound healing, and carry drugs (Kim et al., 2018b; Tsai et al., 2023). We pre-treated the gelatin sponges with Poly-L-Lysine to improve cell adhesion. Research has shown that adding proteins or peptides to biomaterials, such as fibronectin, laminin, collagen, and RGD peptide, can significantly enhance their ability to adhere to cells (Tashiro et al., 1989; Colognato and Yurchenco, 2000; Hersel et al., 2003; Hynes, 2009; Wang et al., 2023). These methods will also be used in our subsequent research.

For the experiment of loading OM-MSCs with gelatin sponge, we selected gelatin sponge cultured for 15 days. It is a rough and relative result, not necessarily the best number of days. Although the gelatin sponge is very large compared to the cells, this regimen does not allow the cells to grow indefinitely. After 30 days of culture, scanning electron microscopy and HE staining revealed that cells in the superficial layer of gelatin sponge were in close contact (Supplementary Figures S2A,B). Too little space between cells will prevent fresh culture medium from transferring substances to cells inside the gelatin sponge, affecting their vitality. Several factors may contribute to the decrease in cell viability, including cell contact inhibition and metabolic waste accumulation. It has been suggested that by changing the shape or volume of the gelatin sponge, or by using 3D suspension culture or dynamic 3D culture, a more ideal biomaterial may be obtained (Hsu et al., 2021; Yang et al., 2021; Liang et al., 2023). It may be better to reduce the volume of gelatin sponge and allow OM-MSCs to be evenly covered in gelatin sponge. We will pursue this further.

A clinical treatment for spinal cord injury inflammation is methylprednisolone, which has completed Phase III clinical trials and has proven to be effective. In the case of acute spinal cord injury, early use of large doses of methylprednisolone can reduce the release of harmful substances in tissues and post-traumatic spinal cord ischemia, thereby minimizing the progression of spinal cord tissue damage. However, corticosteroids cause gastrointestinal bleeding and wound infection (Narum et al., 2014), which is why they are rarely used nowadays. As opposed to this, MSCs are increasingly being used in clinical trials despite many challenges (Galipeau and Sensébé, 2018; Matas et al., 2019). OM-MSCs possess low immunogenicity, can colonize for a long period, and secrete proteins and exosomes that have anti-inflammatory or neuroregeneration effects (Cheng et al., 2014; Zhuo et al., 2024). A variety of nutritional factors can be secreted by MSCs, including vascular endothelial growth factor (VEGF), nerve growth factor (NGF) and hepatocyte growth factor (HGF) (Chang et al., 2014; Chen et al., 2015; Rbia et al., 2019). Nutritional factors promote tissue repair, and studies have shown that MSCs cultured in 3D are more effective at secreting (Kim et al., 2018a; Holkar et al., 2022). In this study, we used gelatin sponge to create a 3D environment for OM-MSCs, which can also promote the secretion of more anti-inflammatory and neurogenic factors.

It is important to evaluate human stem cells in animal models before they can be used in clinical settings. As mesenchymal stem cells (MSCs) express low levels of major histocompatibility complex (MHC) molecules, they are less immunogenic and thus less likely to provoke rejection even after xenotransplantation (Halm et al., 2021). Due to this characteristic, this study treated rats with spinal cord injury using mesenchymal stem cells from olfactory mucosa. In addition, some studies have demonstrated that gelatin sponge materials can be used for a long period of time in animals (Zeng et al., 2023). Clinically used medical gelatin sponge materials were used in this study, and there were no significant changes in the structure of the rat organs following transplantation, which is the main evidence supporting its safety *in vivo*. Despite this, the long-term safety of the delivery material remains to be determined, as it is a mixture of OM-MSCs and gelatin sponge. As a surprise, we found incompletely degraded gelatin sponge material in the GS group without OM-MSCs, but not in the GS + OM-MSCs group. In addition to previous degradation phenomena, such as the material becoming smaller, smoother, and the middle part collapsing in the later stages of culture, we have reason to believe that OM-MSCs can promote the absorption of gelatin sponge. The supporting performance of gelatin sponge also plays a significant role in spinal cord injury, since it is used as a filling material. Tissue repair will be assisted by an appropriate degradation timing. In the future, it will be necessary to study the relationship between gelatin sponge degradation rate and tissue repair.

As well as combining cells and materials, more and more studies are now combining cell derivatives with materials. The MSC-derived secretome, which includes soluble proteins, nucleic acids, lipids, and extracellular vesicles, has shown therapeutic effects similar to MSC transplantation in treating degenerative, inflammatory, and immune-mediated diseases, as well as in tissue repair and regeneration (Ge et al., 2016; Cheng et al., 2014; Ghasemi et al., 2023). The secretome offers similar therapeutic benefits to MSC transplantation but avoids key challenges such as immune rejection, low cell survival, and risks of unwanted cell differentiation (Prado-Yupanqui et al., 2025). The composition of the secretome can be engineered for targeted therapy, and delivery systems can prolong its therapeutic effects (Brennan et al., 2020; Pang et al., 2024; Pang et al., 2025a). MSC-derived secretome provides a cell-free, effective, and safer alternative to MSC transplantation, offering similar regenerative and immunomodulatory benefits while minimizing risks such as immune rejection and low cell survival Its scalability, engineering potential, and broad applicability make it a promising therapeutic strategy for a range of diseases.

In spite of this, this study design still has some limitations. As an example, no single study has been undertaken to determine the cause of pyroptosis of a specific kind of neural cell, and no in-depth study has been conducted on the relationship between neurons, microglia, and astrocytes. Sequencing OM-MSCs loaded on gelatin sponges is also necessary. The differentiation ability of OM-MSCs should be studied, as well as whether the cells in the gelatin sponge have neural markers and differentiation trends. Further studies are needed to assess the safety and efficacy of human olfactory mucosa mesenchymal stem cells in rat models. Moreover, cross-species immune responses may affect stem cell survival and function, an issue that needs to be studied in more depth mechanistically. It is possible to further optimize stem cell transplantation’s method and dosage in the future and combine it with drugs to enhance its efficacy. Meanwhile, developing a more comprehensive evaluation system will facilitate the clinical application of stem cell therapy in spinal cord injury.

## Data Availability

The original contributions presented in the study are included in the article/Supplementary Material, further inquiries can be directed to the corresponding authors.

## Supplementary material

SUPPLEMENTARY FIGURE S1 Identification of cell surface markers and differentiation ability of OM-MSCs. **(A)** Representative images of OM-MSC (Scale bars = 200 μm) **(B)** Flow cytometry assays showing OM-MSCs had high expression of CD73, CD90 and CD105. OM-MSCs did not express CD34 and CD45, purity > 98%. **(C)** Oil rRed O staining showing red lipid droplets in the cytoplasm (Scale bars = 40 μm). **(D)** Alizarin red staining showing red mineralized nodules in the cells (Scale bars = 40 μm).

SUPPLEMENTARY FIGURE S2 The histological changes caused by loading gelatin sponges with OM-MSCs for 30 days. **(A)** HE staining of OM-MSCs after 30 days on gelatin sponges. A dotted line indicates the surface of the gelatin sponge. Scale bars = 100 μm. **(B)** SEM of gelatin sponges loaded with OM-MSCs after 30 days.

SUPPLEMENTARY FIGURE S3 Microglial structure is revealed by TEM. Red arrow: Broken cell membrane. Blue arrows: swollen mitochondria. Scale bars = 1 μm.
