# Supplementary figures and images for: Olfactory mucosal mesenchymal stem cells delivered by gelatin sponge scaffolds promote functional recovery of spinal cord injury
Source: Front Bioeng Biotechnol. 2025 Jul 9;13:1628758. doi: 10.3389/fbioe.2025.1628758 (PMC12283708; doi:10.3389/fbioe.2025.1628758)

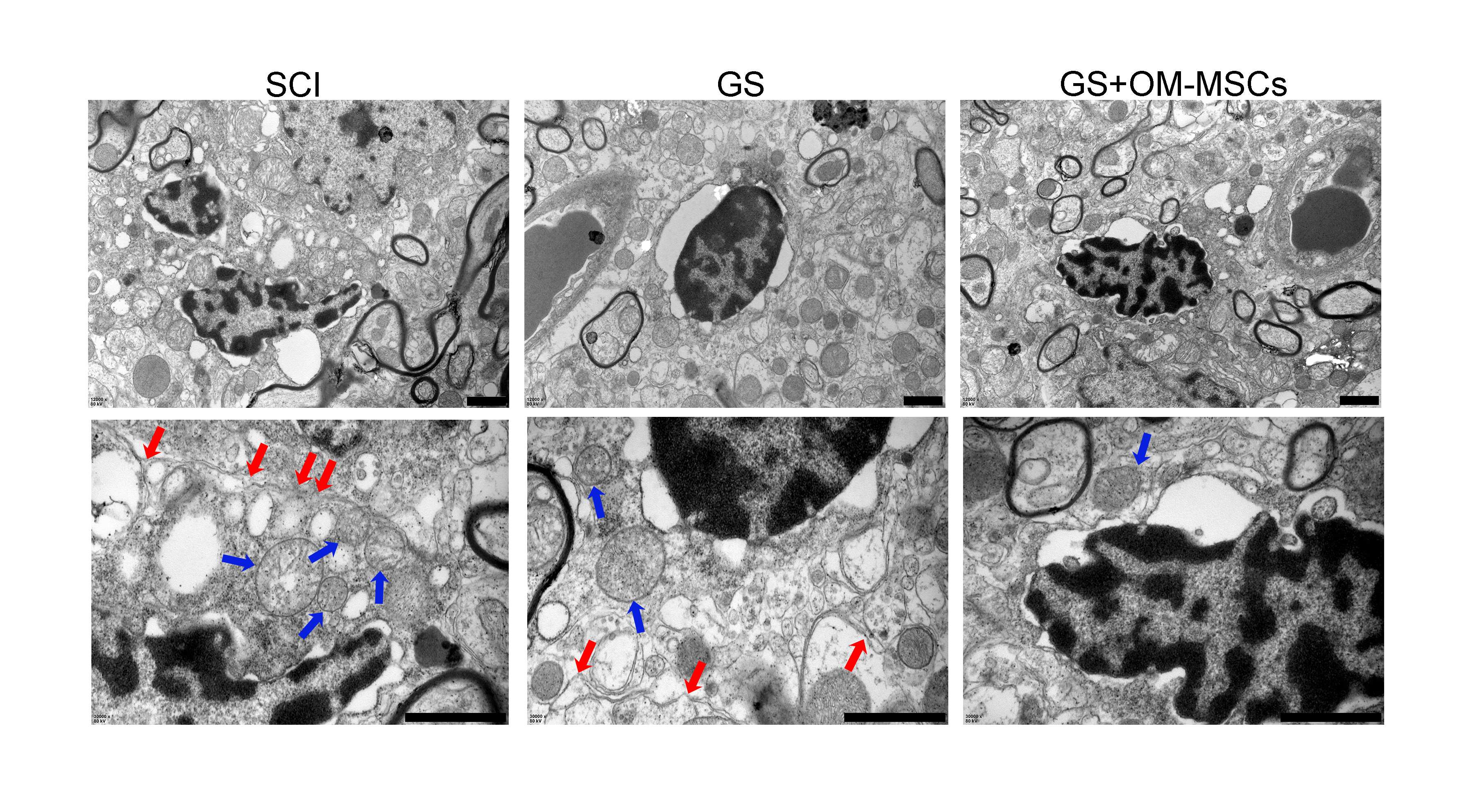

Supplement: Supplementary file 1 [file Image3.tif]

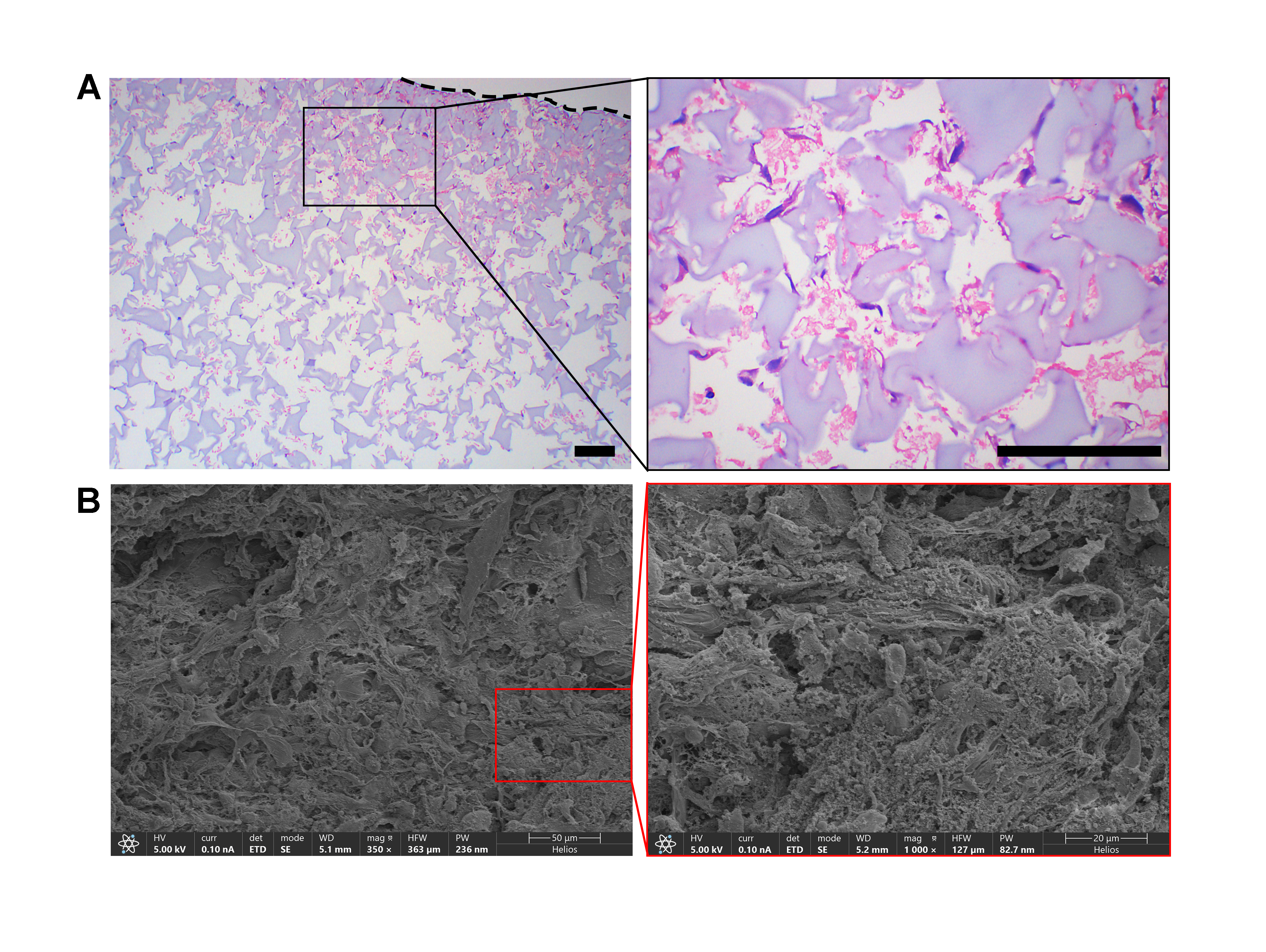

Supplement: Supplementary file 2 [file Image2.tif]

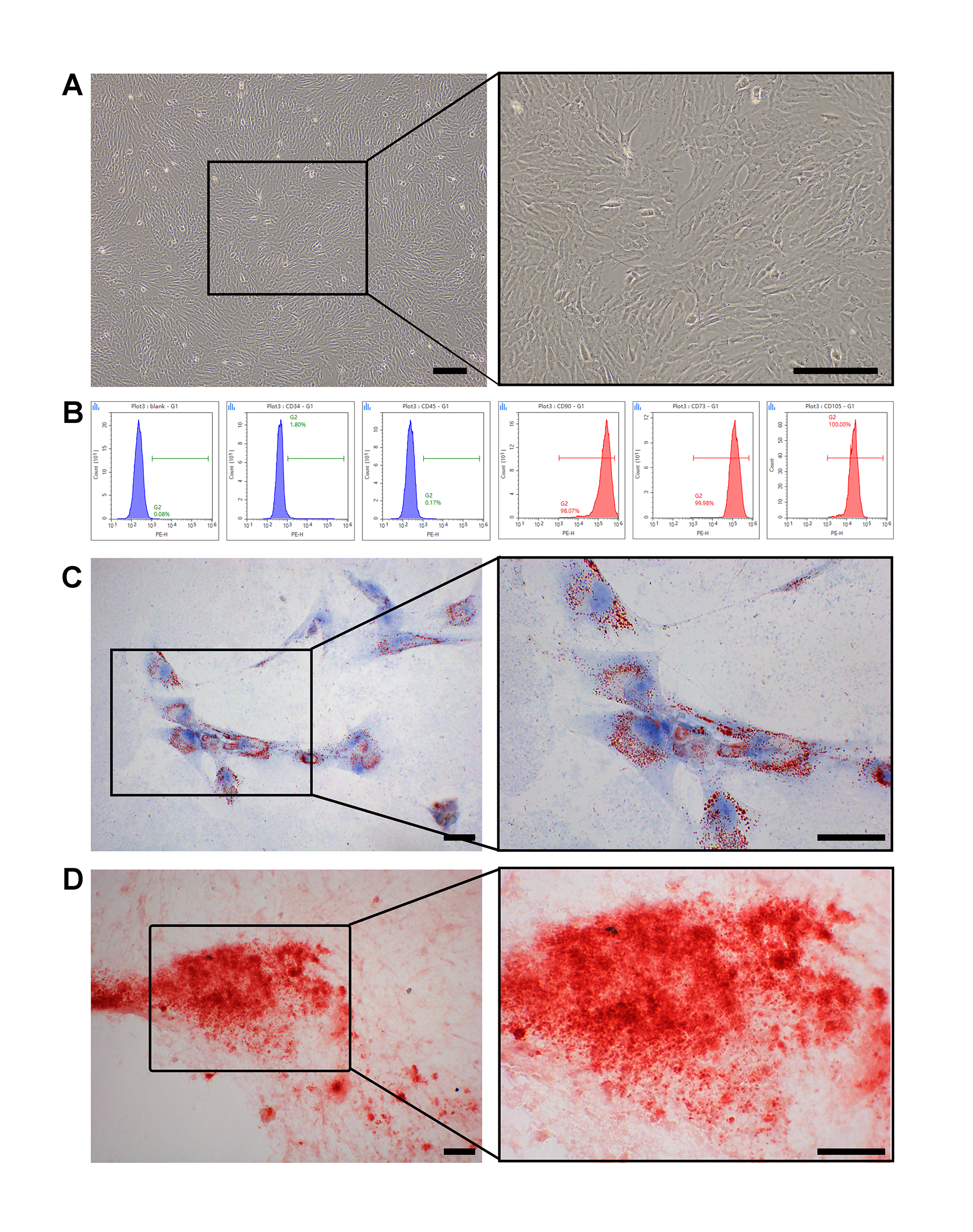

Supplement: Supplementary file 3 [file Image1.tif]
